# Tales from a Hospital Entrance Screener: An Autoethnography and Exploration of COVID-19, Risk, and Responsibility

**DOI:** 10.1177/08912416221131512

**Published:** 2022-10-16

**Authors:** Rachelle Miele

**Affiliations:** 1 Faculty of Social Work, Wilfrid Laurier University, Brantford, ON, Canada

**Keywords:** COVID-19, autoethnography, healthcare workers, risk, responsibilization

## Abstract

This autoethnography explores my experiences as a hospital entrance screener during the first wave of the pandemic in a hospital in Ontario, Canada. In April 2020, I was redeployed from my research role to a hospital entrance screener. Focused on my lived experiences, the purpose of this research is to provide a glimpse into what it was like to work in a hospital early in the pandemic, to understand these experiences in relation to sociocultural meanings, and to try to make sense of my experiences with COVID-19. Through reflections, I offer a critical account of my experiences working as a screener and analyze personal reflections about my thoughts, feelings, and experiences from a post-structural lens. My analysis reveals several themes: responsibilization, risk, emotional labor, policing and securitization, and the hero discourse. My experiences as a screener demonstrate the complexities of the COVID-19 society and experience.

It was in late April of 2020 on a sunny spring afternoon when I received a phone call from the human resource department notifying me that I had been redeployed from my research assistant (RA) position in the intensive care unit (ICU) to a hospital entrance screener position. In Ontario, Canada, a state of emergency was declared on March 17, 2020, due to COVID-19 which included the closure of schools, daycares, libraries, bars and indoor dining, theatres, outdoor amenities, and other nonessential businesses and workplaces (Nielson 2021). There were restrictions on gatherings and Ontarians were told to stay home except for essential reasons (Government of Ontario 2020). In Ontario hospitals, there was a reduction in nonemergent care (Ontario Ministry of Health 2020a) and at the hospital I worked at, some staff deemed nonessential were redeployed while others worked from home. I had been working from home since mid-March when nonessential research was put on hold. My redeployment terrified me. How safe was it to work in a hospital during a pandemic? How can I keep myself and my family safe? Would I get COVID-19? These questions were ever present during my time as a screener.

This autoethnography offers a critical exploration of my experiences as a hospital entrance screener during the first wave of the pandemic in an urban teaching hospital in Ontario, Canada. Focusing on my lived experiences, I set out to answer: what were my experiences as a screener in the early months of the pandemic? In answering this question, I hope to provide a snapshot into what it was like to work in a hospital early in the pandemic and to understand these experiences in relation to sociocultural meanings. To analyze my experiences, I used a poststructural lens, particularly Foucauldian notions of governmentality (Foucault 1998, 2003; Keshet and Popper-Giveon 2018; Rose 2000) and neoliberal health governance (Polzer and Power 2016). From my analysis, several themes emerged: responsibilization, risk, emotional labor, policing and securitization, and the hero discourse. As a snapshot of what it was like to work as a hospital entrance screener, my stories and reflections not only point to the chaos of the situation, they demonstrate the complexities of the COVID-19 society and the experiences of workers.

## Healthcare and Working during COVID-19

The healthcare sector is a high-stress environment and mental health difficulties are widely reported (Karimi et al. 2016). With COVID-19, evidence suggests experiences of burnout (Bettinsoli et al. 2020), increased stress (Cai et al. 2020), increased anxiety (Pan et al. 2020; Yang, Kwak, and Chang 2020), and high rates of symptoms of depression, insomnia, and distress (Lai et al. 2020) are common among frontline healthcare workers. Those who work directly with COVID-19 patients are more likely than other healthcare workers to experience higher levels of depressive and posttraumatic stress symptoms (Di Tella et al. 2020), and overall, experience a deterioration of their mental health (Sasaki et al. 2021). These findings are similar to previous pandemic research (Chan and Huak 2004; Chong et al. 2004; Styra et al. 2008).

Other researchers focus on the conditions of work and the different patterns of risk across healthcare workers. This includes working in an unprepared healthcare system with a lack of planning and personal protective equipment (PPE) (gloves, masks, hand sanitizer, gowns) that put workers at risk (Kalateh Sadati et al. 2021). Issues with PPE supply go beyond having the materials; the lack of PPE available indicates the value of workers and ultimately, their expendability (Willis and Smallwood 2021). For personal support workers (PSW), Rossiter and Godderis (2020) contend that while deemed essential, their care work and their bodies—those of which are primarily racialized women—are invisible. This is because their risks are considered less worthy of protection and consideration. This essentiality and invisibility obscure the risks they experience (Rossiter and Godderis 2020).

Risk extends beyond the healthcare setting and into the home. For healthcare workers, the pandemic disrupts their everyday life and creates fear and anxiety about the transmission of COVID-19 to their families (Kalateh Sadati et al. 2021; Willis and Smallwood 2021). The risk of transmission impacts workers personal relationships and causes isolation from loved ones. For some workers, this meant only seeing friends outside occasionally, while for others this meant moving out of their family home (Willis and Smallwood 2021).

## Theoretical Framework

This study uses a poststructural lens and is guided by a Foucauldian governmentality perspective. Such a lens is well suited to examine my experiences as a screener because it reveals power relations and neoliberal practices of health governance that are found in the social responses to COVID-19. From a governmentality perspective, government is defined as the “conduct of conduct” (Foucault 1991); that is, any activity that aims to guide, affect, or shape the actions or behaviors of persons or a group of people (Gordon 1991; Li 2007). In this sense, the government includes subtle and overt practices that shape and direct the conduct of individuals (Dean 2002; Polzer and Power 2016). As a critical lens, this perspective aims to examine individual choices and behaviors and how those choices and behaviors are regulated at a distance.

In terms of health governance specifically, this lens allows for the examination of neoliberal health discourses and practices. Neoliberalism is characterized by practices that privilege the autonomy of individual citizens. This is done by transforming the role of the state through privatization and the promotion of individualized practices (Rose 2007). For health specifically, this includes public health discourses that promote self-government practices, the redistribution of responsibility of risk management from the state to the individual, and the increasing connection between managing one’s risk and one’s morality (Inda 2005; Polzer and Power 2016). With this lens then, I examine my experiences as a hospital entrance screener.

## Methods, Design, and Analysis

Contributing to the growing literature on COVID-19 and healthcare workers, this autoethnography details my experiences as a hospital entrance screener. As an approach to research, autoethnography looks to analyze personal stories to understand and make sense of social and cultural experiences (Ellis and Bochner 2000; Ellis, Adams, and Bochner 2010). Through storytelling, autoethnographers look both outward and inward, focusing outward on the sociocultural aspects of their experiences and inward at their vulnerabilities, feelings, and emotions (Ellis and Bochner 2000). Focused on reflexivity and voice in social research, autoethnographers reflect, analyze, and interpret experiences in relation to broader sociocultural contexts (Chang 2016; Reed-Danahay 1997; Wall 2006). The intent then of autoethnographies such as this one is to detail and acknowledge the connections between the personal and the social world (Reed-Danahay 2017; Sparkes 2000; Wall 2006).

Part of this process is reflexivity which allows for the examination of how our personal experiences and subjectivities impact our research questions, the research process, data collection, analysis, and interpretation (Aranda 2018; Reed-Danahay 2017; Ristock and Pennell 1996). As such, we must use a critical gaze and take ourselves as the object (Aranda 2018; Green and Thorogood 2014). This research, in part, reflects my social knowledge and location. I am a white working-class cisgender settler woman. I am a sociologist and I research and teach in the area of gender and health. I defended my dissertation just six weeks prior to being redeployed and I worked at the hospital—a teaching hospital located in an urban area—for nine months. As an RA, I worked on a qualitative project that included interviews and focus groups. With the pandemic, qualitative research and funding were put on hold, eliminating my RA position. While it was difficult to move into a position where my skills and knowledge were not utilized, I did not have a choice—I could have refused redeployment, but I was not financially able to refuse work. Given my positionality and research knowledge, my inquiry is greatly shaped by my sociology background.

While I am always a sociologist and a researcher, I did not set out each shift to examine my role or time as a screener. It was not until several months after leaving my position as a screener that I began to reflect on the experience. My data collection and analysis were a reiterative, flexible process (Chang 2016). For the stories I tell below, I draw on reflective journaling pieces I wrote over several months during the third wave of the pandemic in Ontario (between January and April 2021). To write these pieces, I reviewed text message conversations and emails I exchanged with family and friends. These conversations detailed events, my experiences, and my thoughts, feelings, and emotions about the happenings of my job. I also wrote about my memories as a screener, some that I recalled when reading my conversations. Memories are not always seen as a friend to autoethnography, and the truthfulness of memories is often questioned. Despite some issues, memories are significant—we all carry experiences and memories from the past with us as our present selves (Puwar 2021), and as data, memories provide valuable insight on the self (Chang 2016).

Once my reflective pieces were written, I engaged closely with the pieces several times over a period of four months, conducting a reflexive thematic analysis (Braun and Clarke 2019). In analyzing my reflections, I coded by identifying important themes, being sure to note connections to other stories and reflection pieces. Themes were generated by grouping together stories, memories, and feelings that had shared meaning. Reflexive thematic analysis is flexible, and while my theoretical framework was at the forefront of my analysis and readings, I was flexible so that themes that contradicted or were outside of this framework were not ignored. As themes emerged, connections between my experiences, previous literature, and poststructural concepts were made to interpret and understand my experiences.

## Setting the Context: A Day in the Life

Before getting to the analysis, it is important to provide context for my time as a hospital entrance screener. My first shift was on May 4, 2020, from 7:00 pm until 7:00 am. When I arrived, I met my coworker whom I would work every shift with until my final shift which ended at 7:00 am on July 1, 2020. There was no formal training and I learned everything from reading hospital protocols and from my coworkers. We worked 12-hour shifts on a continental schedule (14-day rotation; 2 on, 2 off, 3 on, 3 off, 2 on, 2 off). On day shifts, there were three screeners at our door, and on nights there were two. Few entrances were open throughout the hospital, and some were designated as employee entrances and others as patient/visitor entrances. Our door was the entrance for the helipad, and patients and visitors entered for appointments, to visit dying or deceased family members, or to drop off personal items for those with COVID-19. On day shifts, we saw between 50 and 100 people and at night, a busy shift was about 30 people. Understaffing issues meant that sometimes we were moved to other doors; I once worked at the main entrance of the hospital and screened over 100 people in an hour. Seeing more individuals was worrisome as the number of people I screened increased my risk.

My shifts started with being screened for COVID-19. The door I entered was across from the emergency entrance and there were dots on the ground to ensure that we stayed 6 feet apart, but they had been ruined by the rain and all that remained were circular sticky patches. Just inside the single door, the screening station was located on the left, directly across from the closed coffee shop that I frequented pre-pandemic. The station sounded like an auction house as the screeners raced through the questions. One screener would make sure you were listening by asking if you had recently left Canadian airspace. After being screened, I used the hand sanitizer located just past the station which always burned my hands. I then would go left past the station and down the hall to the mask table to grab my one mask for the day; I would stretch the ear loops before putting it on to ease the pain the mask caused my ears.

With my hands sanitized and my mask on, I walked through the once bustling hallway past a mural that depicted children smiling and riding a carrousel at a carnival. The hallway was usually quiet but often there were nurses and doctors leaning against the wall catching a bit of silence. A doctor in full PPE was once on video chat singing happy birthday to a child. Heading to the elevators, I passed a row of taped-off benches and various units including the ICU. The once busy hallway was quiet—eerily quiet. I found the elevators particularly stressful because of their small, enclosed space so I would try to ride the elevator alone. Out of the elevators, I headed to the screening station past a closed gift shop, a closed flower shop, and the hospital security office. It was here that I would spend the next 12 hours.

I started every shift by reading updates regarding hospital policies, stocking the visitor masks, and refilling hand sanitizer. Some days supplies were limited; on other days, there was no supply. There were shifts when we had one bottle of hand sanitizer at our door and other shifts when we had limited cleaning supplies.

Each screening station was located just inside the door of a hospital entrance. Generally, screening stations consisted of a table (or tables) with plexiglass taped to them. Each station had hand sanitizer, signs listing symptoms, a supply of PPE (gloves, masks, hand sanitizer, wipes, gowns), and a security guard stationed nearby. As a hospital entrance screener, it was my job to screen all individuals who came to our door based on the protocols of that given day. To enter the hospital, patients and visitors had to pass a series of questions about symptoms, their contact with COVID-19-positive individuals, and about their travel.

When I was first redeployed, the newly created hospital entrance screening department consisted of about 150 redeployed hospital employees. Screeners included employees from all areas of the hospital who were researchers, nurses, doctors, administrators, social workers, and various technicians. Managers of the newly created department were redeployed hospital employees too and most began working as screeners. Unfortunately, few managers stayed long in their positions because they were sent back to their pre-pandemic units and jobs.

My station was located at the end of a long footpath and on their way to the station, visitors passed a row of benches that were blocked off with caution tape and a set of tables that were taped off with signs that told visitors that they were not to be used. The final hurdle was walking through an opened-sliding door; our station was to the left and kitty-cornered. Visitors found two screening tables—both were two cafeteria tables pushed together—to their left, my coworker, and to their right, myself. Plexiglass taped to the tables stood between us.

I greeted each visitor the same: “Hi there, I’ll get you to sanitize your hands. Where are you heading today?” followed by the screening questions. It only took a few shifts to memorize the screening questions. Each visitor was given a sticker with the date and the unit they were visiting as well as a mask which we asked them to put on before entering. “Blue to the sky” we would say and “pinch the nose and pull under your chin.”

Throughout the 12-hour shifts, there was a lot of downtime. My coworkers and I exchanged funny stories, talked about television shows and movies, and discussed current events including the Black Lives Matter (BLM) protests that were happening at the time. On night shifts, the only way to survive was to keep yourself busy. I opted to watch movies and play Animal Crossing on my Nintendo Switch. Sometimes my coworker and I would watch a movie together on our separate laptops 6 feet apart.

My family and friends were part of my time as a screener too. It was at the hospital that I met my youngest niece on video chat and watched on Facetime as my oldest niece blew out her candles on her fifth birthday. My mom checked on me each shift to make sure I was wearing my mask and washing my hands. Both of my brothers sent me funny memes while some of my friends chatted via text as late as they could before they fell asleep when I was on night shift. I shared stories and details of my days with them, which made me feel connected despite our distance. They were worried. I can only imagine how worried they all were.

At the end of our shifts, we cleaned our station with hospital-grade sanitizing wipes (a smell I would recognize in an instant if I encountered it again), refilled any low stock, and informed the next shift of any information they needed. Going home was exciting but home felt like a holding spot; home was simply the place I stayed until my next shift. My contract was due to expire at the beginning of July and every shift I completed was one day closer to being with my family. That kept me going.

## Findings

Below are the results of my analysis. Themes include responsibilization, risk, emotional labor, policing and securitization, and the hero discourse.

### Who’s Responsible? The Pandemic Worker

A coworker once described being a screener as a “shitshow”—it was chaotic, confusing, and we were sailing a sinking ship that we were responsible for keeping afloat. As a screener, I was responsibilized for the safety of patients and visitors as well as myself. Both an outcome and symptom of neoliberalism, the responsibility to manage health was individualized. This means individuals are required to take responsibility and are rendered responsible for practices that otherwise would be the responsibility of institutions or states (Rose 1996, 2000, 2007). This meant that the hospital as an institution and the provincial government were not responsible for ensuring enough funding, staff, support, or for having emergency plans already in place. Rather, safety was individualized to us, the pandemic workers.

As screeners, we were responsibilized for creating and maintaining a safe space. When screening stations were first put in place in March of 2020 before I was redeployed, my coworker and a few other screeners found furniture for the station from closed departments throughout the hospital. This included the cafeteria tables and chairs that we used at our station. The hospital provided posters, PPE, and plexiglass on our tables, but we maintained the station:

The screening station was under construction—always. We spent a lot of time trying to make our station as safe or at least feel as safe as we could. We spent a lot of time trying to come up with ways to improve traffic flow, ensure that sanitizer was accessible, and ways to make it easier for visitors to stay 6 feet apart. We would change the angles of our tables, place garbage cans in optimal places for visitors to avoid crowding and to keep visitors away from certain areas, and we added tables to our station to create more distance between ourselves and visitors. We strategically placed hand sanitizing poles along the path to our station: one was placed at the beginning of the footpath and another just inside the sliding doors.

Changes to the physical screening space by the hospital only happened a few times during my contract. We often modified aspects of the station that the hospital put in place:

On a day shift, I arrived to see that dots had been placed on the floor in front of each our stations to indicate to visitors where to stand. They weren’t six feet apart from each other or from our tables—I was not surprised that there was an issue with the dots. We peeled them up and moved them.

We also modified the station by strategically adding furniture. Because our station was kitty-cornered, visitors had to pass by me on my left to enter the hospital; we added a large plastic marketing stand beside my table that previously had been used to hold posters to add more protection around our station. With little oversight, we were responsible for the station and maintaining the environment. In effect, we were responsible for the safety of the space.

Along with the station, screeners were responsible for understanding, interpreting, and implementing policies. The policies regarding screening and procedures regarding visitors changed constantly, and there was a disconnect between policies, how the hospital functioned, and the role of a screener. The following excerpt from my reflections speaks to this experience:

When I first started, visitors could only enter to visit dying family members. To allow visitors to enter, we had to call the unit they were visiting to receive permission for their entry and permission was given by whomever happened to answer the phone. This procedure eventually changed, and permission could only be granted by the manager of the unit. This change caused so many problems—we were often left scrambling to contact the right person and the right one on shift. The hospital directory was poorly kept, and, on many occasions, we were left searching through the hospital website to find who to contact. The visitor policy changed a final time before I left the hospital—managers had to call a newly created visitor hotline with details about individuals who could enter. This information was then put on a spreadsheet for screeners to access but it was often missing information, visitors sometimes did not get added, and there were times when individuals were incorrectly removed.

The ever-changing policies meant that we were met with confused and frustrated patients, visitors, and hospital workers from various units.

There were also the daily COVID-19 numbers. Each day, we received an email detailing the number of patients with COVID-19 and whether they were in the ward or ICU, the number of recovered patients, and the number of patients who had died. I reflected on the numbers:

Numbers—people, not numbers—were used to encourage and push hospital employees. When the number of patients rose, we were scolded; emails reminded us to be diligent, to wash our hands, to keep our distance, and detailed the masking policy. When the numbers went down, employees were thanked and congratulated for their hard work.

The case counts and the emails demonstrate how pandemic hospital workers were made responsible—it was the employees who were responsible for the numbers changing.

Along with daily emails, there was what was known as the “COVID scoreboard.” This was a scoreboard located at one of the employee entrances that detailed the daily case counts at the hospital. One of the managers encouraged us to go visit the door it was at because they believed it was inspiring—which it very well could have been for some. I decided not to visit the door because the thought of keeping score made me uncomfortable. The word scoreboard here is rather telling—it reduces health to a game and mere points. This score keeping dehumanizes patients and reduces individuals to a statistic, while at the same time, positions hospital workers as responsible for winning.

### At Risk and a Risk to Others

The risk of COVID-19 was normal and always present, but the risk itself was not distributed evenly among all hospital workers. As the first contact for patients and visitors, not only was the risk of COVID-19 normal, my exposure to that risk was an expectation of the job. At all times, I was highly aware of the risks of working as a screener which meant that I experienced a heightened level of anxiety. I was hyperaware of the people around me, where they were and how close they were to me, any coughs or sneezes or other symptoms being displayed, and whether they were wearing a mask and doing so properly. I sanitized my hands constantly even though it burned and actively avoided people; I rarely left the screening station even though the station itself was the place I encountered others. The risk was something that I was expected to manage within the walls of the hospital but also beyond.

My level of anxiety increased when a patient entered with COVID-19. The first confirmed patient with coronavirus I saw was transferred to the hospital on my second shift:

It felt like a controlled panic and oddly it felt like I was watching a movie I did not want to watch. There was suddenly a green glow from the helipad and our quiet area of the hospital was filled with the quick steps of hospital security as they made their way to close the elevators, shut down the hallway, and ensure no one was entering or exiting out our doors. I could hear the updates over the security radio giving guards the ETA of the helicopter. Some security waited at the end of the footpath for the helicopter to arrive while a few waited near our station. The bright orange helicopter seemed to appear out of nowhere. With the doors open at the end of the footpath, the helicopter brought a gush of wind that caused our posters, signs, and paper on our tables to flutter. Two paramedics wearing full PPE emerged from the helicopter—one pushed the gurney and the other pulled. Out of the helicopter, the patient was brought down a path and through the opened footpath doors. The gurney made a loud rumble on the metal floor as it was moved up the slight incline. The noise grew louder as they got closer and I wanted to look away, but I sat behind the plexiglass watching; all I could do was watch. Once through our doors, I locked eyes with the patient. I will never forget how terrified they looked.

The pandemic was all-encompassing and the risk of my job spilled over into my personal life. I avoided close contact with others by ordering groceries online, wore a mask outside of my home before it was mandated in Ontario, and did not see any family or friends while working as a screener. I was always aware of the risk I posed to others:

Once when I was taking the elevator down to the lobby of my apartment, a young man on another floor tried to get on with me. With a mask on I shouted, “I work in a hospital!” He slowly backed away from the doors of the elevator.

When I came home from a shift, I changed my clothes and immediately washed them, sanitized my phone, and ensured my hospital shoes were left on a floor mat by themselves. I was always aware of how I was feeling—was I regular tired or COVID tired? Did my throat hurt because I spent 12 hours in a mask talking or was it COVID? What about my headache and are my allergies already bugging me? Home was simply the place I stayed between shifts, and that time felt like an extended lunch break. Inside the walls of my apartment was the only place I knew that I would not be infecting others.

While others posed a risk to me when at work, I was a risk to others outside of the hospital—this impacted my behaviors outside of work. This vulnerability and the need to manage risk highlights the spillover experienced by healthcare workers: they are simultaneously a source of danger and in need of protection (Willis and Smallwood 2021).

### The Impact of Screening

While I had worked in customer service jobs at other times in my life, I was not prepared for the level of care and the emotional labor I needed to perform daily as a hospital entrance screener. As emotional labor—that is, work where I had to manage other’s feelings and my own feelings in a way that was appropriate (Hochschild 1983)—hospital entrance screening was much more than a frontline service job. Instead, hospital entrance screening included emotional work. Related to responsibilization then, not only were we responsibilized in relation to the space and risk, but there was also an aspect of care work. As part of a caring discourse related to hospital workers in general (Boulton, Garnett, and Webster 2021), this labor went unrecognized by the hospital.

One of the difficult aspects of my experience as a screener was witnessing the impact screening and the hospital protocols had on patients and visitors. Most people I interacted with were anxious and nervous about entering the hospital. Some seemed to deal with their nerves by wearing their own mask or wearing various kinds of gloves—this included gardening gloves, kitchen gloves, and one patient wore bright pink gloves with pink feathers.

For many, the process of entering and the protocols caused frustration and sadness, possibly adding to, or creating a stressful health-related experience. I share some of the experiences I witnessed below:

We had visitors come through our doors to say their final goodbyes to loved ones and there were times when visitors were answering the screening questions with tears falling down their face. On my first shift, one man wanted to give his elderly mother who had COVID-19 her bible and rosary, but these items were not essential and not allowed. He stood in front of us crying and said that he just wanted to hug her. A young boy started crying when he realized only one of his parents could go with him to his appointment. I watched as he squeezed his father who had tears running down his face. A mother wept as she hugged her two teenaged children who were not allowed to enter. They were there to say goodbye to their grandmother. A daughter cried when she found out that she could not enter with her father who had dementia for his appointment. A group of siblings who were there to say goodbye to their mother held each other as they sobbed when they found out that she would not be able to see them all together again because only two could enter at a time. A mother begged us to let her in while she cried because her 3-year-old son was having emergency surgery and only one parent was permitted to enter.

Witnessing these moments was difficult and this emotional labor was an everyday aspect of my job. On one hand, I felt immense guilt because I was causing harm. There were instances when implementing and upholding policies was difficult, and when it felt wrong and unfair. On the other hand, working in the hospital, seeing the number of cases, and hearing stories from the ICU and COVID ward meant that I knew how safety was a major concern. To cope, I increasingly became numb to the emotion with each shift and detached myself from the situations:

These emotional moments became normal and while I found these situations to be sad, frustrating, and sometimes heartbreaking, I pushed away my feelings to survive the job. Even as I write this a year later, I still remember the faces of each of these patients and visitors and feel sadness as I recount their stories.

I point these moments out and my feelings regarding these moments not to say that screening or hospital protocols were not needed. Rather, I point these moments out to recognize the consequences and the impact the protocols and my job as a screener had on patients and visitors, to humanize the patients and visitors I interacted with, and the impact this work, particularly as emotional labor, had on pandemic workers. I cannot know the short- or long-term impact of interacting with the healthcare system on any individual that I screened but I can say that there was an immediate emotional impact for many; this is true for myself, too.

### Policing Health

In mid-June 2020, screening shifted in the hospital from a customer service position with mostly redeployed staff (nurses, technicians, social workers, doctors, researchers) to a security job. At this time, the province of Ontario began reopening after the closures since March. For hospitals, this meant that there was a gradual resumption of health services (Ontario Ministry of Health 2020b). In the hospital I worked at, many redeployed screeners were sent back to their regular positions as departments began to reopen—I continued to screen because my position had been eliminated. To fill the screening positions, the hospital recruited and hired those who had completed police foundations or other security training programs. This shift in the screening department was concerning because adding police (including those trained to police) into the hospital securitizes healthcare and is a form of health governance. Securitization includes a form of security policing where institutional practices and laws reflect preemptive police interventions (McCulloch and Pickering 2009; Neocleous 2008). Adding police into healthcare strengthens the role and power of the police—in other words, it extends the role of the police to include defining who should and should not access healthcare. At the same time, this move toward securitization in hospitals during the pandemic also legitimizes policing as a healthcare intervention (Boon-Kuo et al. 2021).

Every new screener I interacted with was suspicious of visitors, patients, and even coworkers including myself. When they screened, it felt like an interrogation and I observed them repeating questions and using an intimidating tone as if they did not believe those they were screening. One screener jokingly referred to unhappy or difficult patients and visitors as “stand up citizens,” while another would get upset if visitors did not know hospital policies. With power and authority as screeners, this profiling-like behavior meant that patients and visitors were analyzed and characterized, and treated accordingly.

One instance involved the screening of a patient who had an Eastern European accent. In this instance, not only was the screener suspicious of the patient, but they were also concerned about controlling who should and who should not have access to the hospital:

When the patient was asked to spell their name, they read the letters off their health card; when the patient was asked their phone number, they read the numbers from their phone. After the patient went in, the cop-screener turned to myself and my coworker and said “Guys, wasn’t that fishy?” Both myself and my coworker shook our heads [in disagreement]. The cop-screener said, “How could someone not know how to spell their name? We should have access to appointments to verify that they should be here.” I explained that we serve an area with lots of immigrants and refugees, and it was likely that English was not their first language. This answer was not good enough for them as they explained that there “could be people coming into the hospital that shouldn’t.” I explained that most people are not looking to hang out at a hospital during a pandemic.

On a rather busy day shift, a new hire denied entry to a South Asian father who was there to accompany his eight-year-old daughter to her appointment. Despite the policy that children could only enter with an adult, the screener assumed that the father was there to translate for his young daughter (translating was not a valid reason for entry). Even when I explained this to the screener after I stepped in and granted the family entry, they insisted that I was following the wrong policy and that I had been deceived by the father. This is an example of profiling—this is concerning because policing in healthcare intensifies existing patterns of criminalization directed toward those made vulnerable, racialized, and minoritized groups (Boon-Kuo et al. 2021).

The suspicion extended to screeners. One of our coworkers drove an e-bike and told myself and a few other screeners about her drive to work the day before:

As usual, we all chatted before the shift change and one of the screeners was telling us this funny story about driving to work the one day on their new e-bike. Immediately after she left—and I mean immediately as in she wasn’t even down the foot path yet—they turned to me and asked “So why does she have an e-bike? Does she not have a license? Or is it a DUI situation?” I was shocked. This told me that they were suspicious of anyone and everyone, including their coworkers.

I was interrogated once too—late in June a visitor who had been visiting his son in palliative care since my first day as a screener tried to enter. With the newly created visitor spreadsheet, his name was not on the list. After sorting this out, one of the new hires confronted me: “how could you let this happen? Who else did you let in that was not on the list?” Luckily, I was in the position to defend myself.

### The Exhausted At-Risk “Hero”

The first time I was thanked for my work as a screener I messaged my mom to tell her about the experience. It was my second week as a screener and an elderly man stopped in front of our screening tables on his way out of the hospital. He was wearing yellow kitchen gloves and leaning on his cane as he spoke. He thanked us for our sacrifices and for all the important work we were doing. I smiled under my mask and told him to have a great day.

In the lobby of my apartment after a day shift, a group of young adults noticed my hospital badge while we waited for the elevator. They let me take the elevator first because of all the work I was doing.

These experiences were awkward, common, and I never knew how to respond. My coworker and I would always comment to each other when we were thanked that we were not heroes—can you be a hero if you do not want to be there?

Frontline healthcare workers (mostly directed toward nurses and doctors) have been labelled “heroes” by politicians, the public, and mass media (Boulton et al. 2021; Einboden 2020), constructing the hero discourse. In the early months of the pandemic in Ontario, the public took to their porches and balconies to bang pots and pans to show support for healthcare workers; corporations created commercials and marketing materials thanking frontline staff; and politicians referred to healthcare workers as heroes (Mohammed et al. 2021). My social media was filled with posts thanking healthcare heroes, and lawn signs thanking frontline workers popped up all around the city. We were thanked for our sacrifices and idealized for our selflessness by patients, visitors, and hospital leadership.

While the hero discourse is presented as neutral, it has social and political implications as it hides the realities of being a frontline healthcare worker and my reality as a screener:

There were mask shortages. We were told to limit the number of masks we used to one a day unless necessary and to reuse them after we ate. There were limited human resources, personnel shortages, and high turn around for leadership roles because of deployment. There were shortages of hand sanitizer and cleaning supplies. There was a disconnect between policies and screening. There was a lack of infrastructure for data entry and dealing with visitors. The job was risky, I was always exhausted, and I was isolated from friends and family. I was not valued like a hero. *I* was expendable. My job was deemed essential, but *I* was not—my body and its safety were secondary.

I was involuntarily placed in this role, and I had to endure the risk to survive economically. The construction of the hero discourse meant that I was expected to overcome challenges and preserve because I was a hero. This discourse hides the realities of working as a screener and in doing so, works to further individualize healthcare workers, lessening the responsibility of hospitals and the government while at the same time normalizing risk (Mohammed et al. 2021).

## Final Thoughts and Conclusions

While I was only a screener for a short time, my experiences reveal a great deal about what it was like to work in a hospital early in the pandemic in Ontario. The hospital struggled to deal with COVID-19, and overall, the Ontario healthcare system revealed itself to be fragile, unprepared, underfunded, and understaffed (Einboden 2020). Reinforcing neoliberal approaches to health, within the hospital the virus was individualized, lessening the responsibility of the government to legislate systemic changes. Instead, individuals like myself were charged to act and to heroically overcome the faults of the healthcare system (Mohammed et al. 2021). This meant that while I was called a hero by politicians and the media, my body was put at risk—but it was much more than this; I was a threat to others because of the risk of my job. This duality of managing this risk resulted in negative spillover into my life at various levels, increased anxiety, isolation from family and friends, and caution that encompassed all aspects of my life (Willis and Smallwood 2021).

Hospital screening was much more than a newly created position—screening possibly caused harm to visitors, patients, and screeners. This included the enforcement of policies and practices that impacted those already dealing with difficult and stressful health-related moments during a pandemic. This is especially concerning for women; those with disabilities; racialized and minoritized individuals; those experiencing economic insecurities; 2SLGBTQIA+ individuals; and those with several of these identities, all of whom report negative, discriminatory, and harmful experiences interacting with the Canadian healthcare system (see Goodman et al. 2017, Schwab et al. 2022 for examples). The presence of screeners with a policing background is also concerning. Not only does it move the healthcare system away from public health, it also strengthens the role of the police to define who is a threat and who is not (Boon-Kuo et al., 2021). This has implications for healthcare access and strengthens existing patterns of criminalization directed toward racialized and minoritized groups (Boon-Kuo et al. 2021).

After reflecting on my experience, I wonder about other pandemic workers and workers labeled essential in healthcare and in other sectors. What was their experience like with risk? How did they cope with issues with PPE, responsibility, and spillover? These are all important questions for research to consider and answering such questions will provide insight into the complexities of the pandemic society and neoliberal health discourses, practices, and norms.

On my final shift at around 2:00 am, my coworker and I ordered pizza to celebrate the end of my time as a screener. We watched one final movie together on our laptops 6 feet apart. When I left the hospital for the final time, a weight was lifted as I knew I could finally hug my nieces, mom, and brothers soon. I was privileged to be able to leave the hospital and my screening job, and I know I was simply replaced by another hero, someone exposed and put at risk just as I was.
